# Therapeutic Approaches to Target Cancer Stem Cells

**DOI:** 10.3390/cancers3033331

**Published:** 2011-08-15

**Authors:** Arlhee Diaz, Kalet Leon

**Affiliations:** Department of Systems Biology, Center of Molecular Immunology, 216 Street, PO Box 16040, Atabey, Havana 11600, Cuba; E-Mail: kalet@cim.sld.cu (K.L.)

**Keywords:** CSC, CD133, angiogenesis, EGFR

## Abstract

The clinical relevance of cancer stem cells (CSC) remains a major challenge for current cancer therapies, but preliminary findings indicate that specific targeting may be possible. Recent studies have shown that these tumor subpopulations promote tumor angiogenesis through the increased production of VEGF, whereas the VEGF neutralizing antibody bevacizumab specifically inhibits CSC growth. Moreover, nimotuzumab, a monoclonal antibody against the epidermal growth factor receptor (EGFR) with a potent antiangiogenic activity, has been shown by our group to reduce the frequency of CSC-like subpopulations in mouse models of brain tumors when combined with ionizing radiation. These studies and subsequent reports from other groups support the relevance of approaches based on molecular-targeted therapies to selectively attack CSC. This review discusses the relevance of targeting both the EGFR and angiogenic pathways as valid approaches to this aim. We discuss the relevance of identifying better molecular markers to develop drug screening strategies that selectively target CSC.

## 1. Introduction

Solid tumors are abnormal masses of highly complex and heterogeneous tissue that include tumor cells, stroma, inflammatory infiltrates and complex vascular structures. The cellular origins of most solid tumors are largely unknown, but it has long been speculated that different subtypes may reflect distinct cells of origin at the time of tumor initiation. In recent years, the CSC model of tumorigenesis has received increasing attention to account for heterogeneity and inherent differences in tumor-regenerating capacity. The term of CSC refers to a subset of tumor cells with the ability to self-renew and generate the diverse set of cells that comprise the tumor. For that reason, CSC constitutes a reservoir of self-sustained cells with the exclusive ability to self-renew and maintain the tumor. Furthermore, they could constitute a population that is intrinsically resistant to current therapies designed to kill rapidly dividing cells due to their inherent ability to enter the cell cycle infrequently, surviving treatments without major damage and being associated with tumor relapses in patients. Together these properties ensure a sustained tumorigenesis for CSC emerging as a primary therapeutic target for current anticancer therapies.

However, in contrast to what has been reported for hematological malignancies, the existence of a rare population of cancer cells exhibiting stem cell-like characteristics and promoting tumor growth in solid tumors remains controversial [1,2]. Several issues of paramount importance, like a stringent definition of CSC or the lack of appropriate specific markers that only target cells that meet the criteria for a CSC, remain unclear for solid tumors. Moreover, the translation from the theoretical benefit of CSC eradication into its actual clinical benefit has to be experimentally demonstrated.

Therefore, if the CSC hypothesis holds true, we can predict that the complete eradication of this population may be curative, at least in tumor types where neoplastic growth and differentiation depend on CSC. Although the clinical relevance of CSC remains an open question, preliminary findings suggest that specific targeting may be possible.

In this review we discuss the challenges of some of the currently used markers to identify CSC. In addition, we discuss the main mechanisms of resistance of CSC to current therapies and finally provide an update in current clinical trials and new perspective targets for addressing CSC in the clinics.

## 2. CSC Markers

Early evidences for the existence of CSC initially came from studies done in the 1990s in acute myelogenous leukemia (AML) [3,4]. These studies demonstrated the existence of a subset of leukemic cells responsible for AML in immunodeficient mice that displayed a similar cell surface immunophenotype as normal hematopoietic stem cells [3]. However, it took six additional years until CSC were first isolated from a solid malignancy, when Al-Hajj *et al.* described a breast cancer cell population harboring a CD44^+^CD24*^−/low^* immunophenotype with enhanced tumor-initiating capacity [5]. Subsequently, CSC-enriched populations were prospectively isolated from many other human malignances, including those arising from brain cancer, melanoma, colorectal cancer, and prostate cancer, among others [6-10].

Thus far, all the above-mentioned studies have been performed using cell surface molecules as instrumental tools in identifying CSC subpopulations. Cell surface markers have proved to be useful in the isolation of subsets enriched for CSC, comprising a large list of molecules that includes CD133, CD44, CD24, epithelial cell adhesion molecule (epCAM), THY1 and ATP-binding cassette B5 (ABCB5), as well as Hoechst_33342_ exclusion by the side population cells. Amongst the above-mentioned markers, CD133 and CD44 have undergone the most extensive research, proving potential tools for therapeutic approaches.

### 2.1. CD133

The CD133 molecule (also known as prominin-1) is currently one of the most popular markers employed to define CSC populations. Specifically, the expression of prominin-1 protein in adult humans is not limited to the stem and progenitor cells [11], but it is also expressed in epithelial cells [12]. In contrast, the expression of AC133, the glycosylation-dependent AC133 epitope of human prominin, appears to be restricted only to a subset of molecules, such as those specifically expressed in hematopoietic stem and progenitor cells [13] and cells dedifferentiating in the process of malignant transformation [12]. Therefore, it is important to notice that AC133 antigen is not synonymous with human CD133. Only the AC133 is down-regulated upon cell differentiation, whereas the expression of CD133 is independent from cells' state of differentiation [12]. For that reason, it is likely that AC133, but not CD133, is a reliable CSC marker. Accordingly, the majority of studies outlined in this section refer to studies that detected CD133 by its glycosylation epitope, AC133; but one has to be cautious when interpreting results from experiments where it is unclear if the antibody detected CD133 or AC133.

Initial studies ascribed a functional role to CD133 as an organizer of the plasma membrane topology, dictating interactions with cholesterol and maintaining an appropriate lipid composition within the plasma membrane [14,15]. However, expanding evidences have recently highlighted the role of CD133 as a marker of CSC in various human tumors, including lung, prostate, pancreatic, and colorectal carcinomas, among others [16-18].

Nevertheless, most of the accumulated research for establishing the role of this molecule as a marker for CSC comes from studies done in brain tumors: CD133 has been found to mark CSC in different types of brain tumors, including glioblastoma multiform (GBM), pediatric medulloblastoma and ependymomas [6,19-22]. Moreover, CSC with dual expression of CD133 and the early lineage marker nestin have been isolated from several human brain tumors (including medulloblastomas, glioblastomas, and oligoastrocytomas) [21-25]. CD133^+^ cells, in contrast with their CD133^−^counterparts, have shown an ability to self-renew, undergo multi-lineage differentiation (to neurons, astrocytes, and oligodendrocytes *in vitro*) and recapitulate the original tumor phenotype *in vivo*. In addition, Marzesco *et al.* have supported a potential functional role of CD133 in the maintenance of a stem/progenitor cell state in neural progenitors and other epithelial cells [26]. The authors showed the existence of small CD133-containing membrane particles in the ventricular fluid within the developing embryonic mouse neural tube and adult human tissues, whose appearance coincided with changes on the embryonic neuroepithelial cells, such as the regression of microvilli and the formation of large pleomorphic protuberances [26]. Moreover, these particles were released by the epithelial model cell line Caco-2 upon differentiation [26]. Altogether, these preliminary observations highlight a functional role of CD133/prominin-1 in sustaining a stem cell phenotype, and support this molecule as a central target for successful cancer therapy.

However, despite preliminary findings, solid evidence supporting the role of CD133 as predictor of patient outcome is still required, an issue that remains unclear for most, if not all CSC markers. In line with this, a recent study has provided the first evidence that CD133 expression in glioma may predict a poor patient survival whereas the proportion of CD133^+^ cells appears to be an independent risk factor for tumor relapse and speed of tumor progression [27].

### 2.2. CD44

Similarly to CD133, the surface marker CD44 represents one of the most popular CSC markers. CD44^+^ populations have being found to be enriched in breast, prostate, head and neck, and colon tumors among others [5,7,8,28]. Although the specific role of this molecule in CSC biology remains poorly understood, it is well known that it binds several ligands and it is involved in cell adhesion and signal transduction processes [29]. More importantly, the CD44 molecule constitutes a cell surface marker that could represent a potential target for specific monoclonal antibodies-based therapies.

Previous findings establishing a role for CD44 as a CSC marker emerged from preclinical studies done in AML. In such studies, a putative CSC subpopulation has been described to express a CD34^+^CD38^−^ phenotype, in addition to high levels of CD44 [30]. In that report, the *in vivo* administration of a monoclonal antibody against the CD44 molecule reduced leukemic repopulation in immune-deficient mice transplanted with human AML. Additionally, that study suggested that an interaction between AML leukemic CSC and the microvascular niche is required to maintain their stem cell properties [30]. Therefore, this therapeutic approach has supported a role for CD44 as a key regulator of AML tumorigenic properties and provided a paradigm for targeting CSC niches.

On the other hand, several efforts have been made in order to establish the precise role of CD44 as a prognostic marker in cancer patients. However, despite a few clinical studies that have shown encouraging results, there are controversial findings indicating the need of further evaluations to confirm this. For example, a large study that examined a 186-gene invasiveness signature generated from CD44/CD24^−/^*^low^* tumorigenic breast-cancer cells or normal breast epithelium, indicated its strong association with a reduced overall survival and a propensity of tumor to metastasize [31]. In contrast, in another study that evaluated 136 paraffin-embedded clinical specimens, no correlation was found between the prevalence of CD44^+^CD24^−^ tumor cells and clinical outcome or survival [32], arguing against the significance of this phenotype in identifying cells with tumorigenic potential in human breast cancer [5]. Nevertheless, the same study did indicate that the prevalence of CD44^+^CD24^−^ tumor cells might favor metastatic disease [32]. These findings were further confirmed in three other tumor types including medulloblastoma, lung cancer, and prostate cancer [31].

### 2.3. Unresolved Issues Related to the Study of CSC Markers

More refined research is therefore mandatory for the accurate identification and isolation of appropriate CSC markers representative for such population. An important issue to be addressed by future research is connected to the phenotypic heterogeneity within CSC populations. In glioma, for example, CD133 expression does not always appear to characterize the CSC subpopulation. A study published in 2007 by Beier *et al.* addressed the tumorigenic potential of glioma subpopulations with different molecular profiles [20]. Interestingly, CD133^−^ and CD133^+^ subpopulations showed a similar ability to form tumors in nude mice, despite displaying a distinct molecular gene expression profile, thus suggesting that glioblastoma-derived CSC may have different cells of origin [20]. More recently the same group confirmed the existence of two subgroups of GBM: Type I CSC lines that display “proneural” signature genes and resemble fetal neural stem cell lines, and type II CSC lines that show “mesenchymal” transcriptional profiles similar to adult neural stem cell lines [33]. This result suggests that the heterogeneity of GBM may derive from different types of originating CSC. Interestingly, type I CSC lines showed a CD133^+^ phenotype and formed neurospheres, whereas type II CSC lines, displayed semi-adherent growth and lacked CD133 expression [33].

Moreover, some overlap is evident between the cell surfaces phenotypes of CSC described so far by different groups for the same tumor type. In colon cancer, an overlap for EpCAM*^hi^*/CD44^+^ [8] cells and a minor proportion of the CD133^+^ population has been found [16]. Similarly, a small overlap between CD133^+^ [34] and CD44^+^/CD24^+^/ESA^+^ [17] populations has been reported in pancreatic cancer. Such variations may be attributable to different culturing conditions before cell sorting. In glioma, for example, the expression of CD133 might change during long term culture in serum-containing medium [35]. We have recently found that a rapid morphological change from adherent to non-adherent tumor spheres is observed in U87MG human glioma cells after serum depletion conditions. These non-adherent tumor cells posses the capability to self-renew and to form secondary spheres derived from single parental cells after repopulation in serum-free neural stem cell culture medium. Similar observations have also been reported by Yu *et al.* [36]. Additionally, these cells have the ability to produce three different types of progenies, and to form secondary tumors pathologically different from tumors formed by U87MG cells when xenografted in nude mice [36]. Thus, the above preliminary findings coming from different groups are likely to confirm the high susceptibility of CSC to modifications in cell culture conditions.

## 3. Mechanisms of Resistance of CSC

The existence of CSC has a crucial implication for cancer biology and therapy and its eradication should be critical in achieving disease control. Nevertheless, although CSC are particularly resistant to conventional therapies, the precise mechanisms by which CSC resist classical therapeutic interventions, such as cytotoxic chemotherapies or radiotherapy-induced cell killing, remain elusive to date. Some of these mechanisms include slow cell cycle kinetics, relative resistance to oxidative or DNA damage, efficient DNA repair, and intrinsic expression of anti-apoptotic molecules [19,37-39]. Other mechanisms of CSC resistance to cytotoxic agents involve the high expression of multidrug-resistance-type membrane transporters, residing in hypoxic niches, and the expression of specific drug-detoxifying enzymes [40-44].

### 3.1. Modulation of Cell Cycle Kinetics

It has been postulated that resistance to conventional therapies by CSC may be due to their concept that these cells may enter a quiescent state [37]. Accordingly, quiescent CSC are thought to be more resistant to cytotoxic therapies, whereas rapidly dividing cells have long been known to be more sensitive to such therapies. This idea has been well documented in at least a subset of leukemia stem cells that are quiescent and appeared to protect leukemic progenitor cells from therapeutic actions [37,45], but remains poorly understood for solid malignancies. Indeed, controversial findings published in this field have led researchers to argue that current results are not conclusive. In the study published by Al-Hajj *et al.* in 2003, in which CSC were isolated for the first time from a solid malignancy, cell cycle profiles of human breast CSC and non-tumorigenic cells were found to be identical, arguing against large difference in cell cycle status between both populations [5]. In contrast, other studies show that both normal and malignant human CD44*^high^* epithelial cells with stem-like properties are more resistant to the induction of apoptosis than their CD44*^low^* counterparts [46], and such differences are directly associated with an extended G_2_ phase of cell cycle in those cells exhibiting the CSC phenotype. Furthermore, the targeting of G_2_ checkpoint proteins releases these cells from the G_2_ block and sensitizes them to apoptosis, suggesting a useful approach for cancer therapy [46].

### 3.2. Efficient Mechanisms of DNA Repair

Several reports have linked the radioresistance of CSC to their ability to more efficiently repair DNA damage induced by ionizing radiation than non-CSC. These studies have associated the resistance of CSC to a specific mechanism, investigating the DNA damage response in matched CD133^−^ and CD133^+^ cells derived from gliomas [19]. Findings from such studies suggest that ionizing radiation is able to induce DNA damage to similar degrees in CD133^+^ and CD133^-^ cells, but CD133^+^ cells have the ability to repair the DNA damage more efficiently and underwent less apoptosis than their CD133^−^ counterparts [19]. Through this research, several kinases have been identified as being involved in these processes of double strain DNA break repairmen, including the checkpoint kinases, CHK1 and CHK2. CHK1 and CHK2 kinases are preferentially activated in CD133^+^ but not CD133^−^cells in response to DNA damage caused by ionizing radiation and some chemotherapeutic agents [47]. Therefore, we can predict that CD133^+^ cells may become more sensitive to the action of ionizing radiation by using an inhibitor of the checkpoint kinases CHK1 and CHK2. Altogether, these results suggest that the use of drugs that target checkpoints kinases CHK1/2 may have important biological implications for cancer treatment.

### 3.3. Intrinsic Expression of Anti-Apoptotic Molecules

Activation of the apoptotic program has been shown to be responsible for chemo- and radiation-induced cytotoxicity in tumor cells, while alterations in the apoptotic machinery have been related to resistance in several tumor types [48]. Resistance to apoptosis in CSC may therefore depend on abnormalities of the cell death pathway, such as overexpression of anti-apoptotic factors or silencing of key death effectors. Resistance to therapy might be conferred to CSC through the activation of the Akt pathway [49] and the overamplification of apoptosis inhibitor proteins. In addition, chemoresistant hepatocellular carcinoma CSC were found to preferentially activate Akt/PKB and Bcl-2 cell survival pathways [50]. Moreover, inhibition of Akt by perifosine sensitizes CSC to radiation-induced apoptosis [51]. In addition, a study undertaken in primary cultured cell lines established from GBM patients provided evidence that, in CD133^+^ CSC, the chemotherapy-induced resistance is due, at least in part, to the higher expression of anti-apoptosis proteins, including Bcl-2, FLIP and BCL-XL, as well as the inhibitor of apoptosis protein (IAPs) families (XIAP, NAIP and surviving) [24]. Moreover, several studies indicate that increased levels of the anti-apoptotic protein Bcl-2 correlates with chemotherapy resistant disease and decreased overall survival.

Taken together, these observations suggest that characterization of Akt and Bcl-2 expression in CSC may have clinical significance. In this regard, the inhibition of FMS-like tyrosine kinase 3 (FLT3) receptor signaling, an important hematopoietic growth pathway upstream of Akt, has shown encouraging results in a phase II clinical trial conducted in patients with AML [52].

Moreover, several multiphase clinical trials are currently evaluating the optimal dose and the efficacy of AEG35156, an inhibitor of the XIAP isoform, in combination with cytotoxic agents in advanced solid tumors. These studies might provide another promising pathway for therapeutic intervention targeting the apoptotic regulation of CSC.

### 3.4. Relative Resistance to Oxidative or DNA Damage

A paper published by Diehn *et al.* in 2009 suggested a direct link between the existence of CSC and their role in mediating radiation resistance by the modulation of the levels of reactive oxygen species (ROS) [39]. In that report, the authors demonstrated that subsets of CSC in some human breast tumors may contain lower intracellular ROS levels than corresponding non-tumorigenic cells, consistent with ROS being critical mediators of ionizing radiation-induced cell killing. Additionally, CSC in those tumors, defined by the CD44^+^CD24*^−/low^* Lin^−^ or Thy1^+^CD24^+^Lin^−^ phenotypes, developed less DNA damage and were preferentially spared after irradiation compared to non-tumorigenic cells. More important, the pharmacologic depletion of ROS scavengers in these cells significantly decreased their colony forming ability in culture, and finally led to radiosensitization of the Thy1^+^CD24^+^Lin^−^CSC-enriched population. Therefore, low levels of ROS in some CSC appear to be at least partially due to the increased production of free radical scavengers, providing an alternative explanation for resistance to radiation in CSC-enriched populations [39].

### 3.5. Residence in Hypoxic Niches

Accelerated cellular expansion leads to the formation of intermittent regions of hypoxia within the tumor bulk and the acidification of the microenvironment, by an increased aerobic glycolysis, which in turn results in the release of several proteins involved in the response to such cellular conditions [53]. The hypoxia-induced factor (HIF)-1, is a transcription factor stabilized in normal cells by hypoxic conditions that has been demonstrated to increase the neovascularization and has been suggested as a factor that regulates tumor radioresistance in a variety of solid tumors [54]. CSC have been shown to have a molecular phenotype similar to that of cells exposed to hypoxic conditions [55]. Particularly, they also have been shown to express vascular endothelial growth factor (VEGF), a molecule related to the induction of neovascularization [21]. However, it is not clear whether such hypoxic phenotype expressed in CSC is a consequence of their chronic exposure to hypoxic conditions, or to the genetic or epigenetic modifications related to the malignant transformation. It is well expected that CSC located within tumors might be chronically exposed to hypoxic conditions. Nevertheless, recent reports in the literature have shown that they might be also found along perivascular areas, expressing a “hypoxic phenotype”, without any evidence of hypoxic stress [56,57]. Whether or not hypoxic conditions are directly related to the malignant transformation of normal stem cells remains to be elucidated.

On the other hand, the up-regulation of the epidermal growth factor receptor (EGFR), another molecule typically up-regulated in CSC, [58] has been reported to be directly linked to HIF-1 activation, given an alternative explanation for the lack of receptor mutations in human tumors that overexpress the EGFR protein [59]. Remarkably, tumor hypoxia, like EGFR expression, has been considered a predictor of tumor progression, resistance to radiotherapy and poor clinical outcome in patients, establishing a close interplay between the two [58,60].

### 3.6. High Expression of Multidrug-Resistance-Type Membrane Transporters

Multidrug resistance appears to be inherent to putative CSC and has also been involved in refractoriness to several cytotoxic drugs, including gemcitabine, cisplatine and cyclophosphamide [61-63]. Classical multidrug resistance is attributed to an elevated expression of ATP-dependent drug efflux pumps belonging to the superfamily of ATP-binding cassette half-transporter proteins ABCG2 and ABCG5, and multidrug resistance protein 1 MDR1 (also known as ABCB1) [40]. Drug efflux mediated by ABC transporters leads to a decreased cellular accumulation of anticancer drugs and it is considered a major setback of currently applied chemotherapeutic regimens. Accordingly, high expression of the drug transporter and chemoresistance mediator ABCB5 has been correlated with melanoma progression, and specific targeting of the ABCB5^+^ population with a monoclonal antibody significantly inhibited tumor growth in patients [64].

In addition, the functional similarities between CSC and normal stem cells predict that many pathways or molecular markers that are specifically overexpressed by CSC compared to non-tumorigenic cells, will also be found to be activated or overexpressed in normal stem cells compared to their more differentiated counterparts, thus representing an additional barrier to developing effective and less toxic anticancer therapies based on the targeting of CSC. Theoretically, it is predictable that many potential CSC-specific therapies may induce significant normal tissue stem cell toxicity, similar to current standard cytotoxic therapies. This is fully in line with the emerging insight that potential CSC-specific therapies should be able to target uniquely CSC, sparing normal stem cells.

Therefore, based on accumulating evidences, it appears that CSC may resist standard cytotoxic therapies through a combination of mechanisms, which may be unique to a given tumor. Therefore, a better comprehension of the mechanisms operating in each specific tumor will be critical to understand how CSC resists the available standard therapies and should help us to better design future protocols of chemo and radio-sensitization.

## 4. Therapies Targeting CSC

The CSC hypothesis holds the idea that specific therapies against CSC may lead to develop curative strategies, or at least to avoid common relapses. Based on this hypothesis, new therapies should be oriented towards the eradication of the minority population of CSC, instead of the rapidly dividing but terminally differentiated cells, which constitute the bulk of the tumor. However, the contribution of CSC in cancer development is still unclear, and solid evidence of benefits derived from such therapies are relatively low. Nevertheless, due to the well demonstrated fact of the inherent resistance of CSC to cytotoxic therapies; it is predictable that the specific targeting of such population should result in a better control of the disease. Therefore one might expect that combinatorial treatments involving both cytotoxic and targeted therapies, including those against CSC, will probably require ablating all cancer cells. It is thus likely that the same approaches to develop therapies against molecular targets based on the use of monoclonal antibodies—and small molecules—may also be usefully applied for anti-CSC therapies. These approaches include the targeting of CSC via monoclonal antibodies against cell surface molecules (CSC markers), targeting CSC via developmental stem cell pathways and targeting CSC via CSC microenvironment. An update of clinical trials assessing these targets is illustrated in Table 1 [65].

### 4.1. Therapies Targeting CSC Surface Molecules: The EGFR Molecule

The EGFR is a well-known validated target for molecular anticancer therapies [66]. This molecule is commonly overexpressed in several epithelial cancers, and its overexpression has largely been correlated with poor prognosis in patients [67]. Indeed, several classes of EGFR antagonists, such as monoclonal antibodies, small tyrosine kinase inhibitors, or antisense oligonucleotides have been developed and are under evaluation alone or in combination with cytotoxic therapies in patients [68].

One example of new EGFR antagonists with a well established antitumor activity is the humanized monoclonal antibody nimotuzumab (CIMAher^®^, h-R3) [69]. Nimotuzumab has shown encouraging results in the treatment of patients with head and neck and brain tumors, when used in combination to radiation therapy [70,71] or cytotoxic drugs [72]. Previous studies in our group have also revealed the superiority of combining nimotuzumab and radiation in the treatment of human GBM U87MG cells xenografted in mice [73]. More interesting, this study outlined the ability of anti-EGFR antibodies to target the CD133^+^ CSC population, acting as radiosensitizers in this tumor model. Remarkably, recent studies have revealed that CSC markers nestin and CD133 are co-expressed alongside EGFR on the cell surface of the U87MG cells providing a plausible explanation for such findings.

Precise mechanisms by which anti-EGFR antibodies may radiosensitize tumor cells are not fully understood, but their ability to target the CSC population is one likely mechanism. A possible explanation is that the abrogation of EGF may prevent CSC from entering in a more terminal state of differentiation, which is in line with previous findings [74]. This hypothesis might explain why some molecular-targeted therapies produce mostly a cytostatic, rather than a cytotoxic effect [75] which in turn would explain the induction of stable disease without evidences of tumor shrinkage [76]. Furthermore, EGFR antagonists may potentiate the activity of ionizing radiation by forcing most of the cells to enter into the more sensitive phase of the cell cycle G_0_/G_1_ [77].

So far, it is known that all the anti-EGFR monoclonal antibodies with therapeutic activity share the property of blocking the binding of EGF to EGFR, subsequently inhibiting EGFR-related downstream pathways [73,78,79]. Moreover, the EGF is a mitogen that seems to be necessary for harvest tumor cells with a CSC phenotype [74]. Accordingly, tyrosine kinase inhibitors AG1478 (Calbiochem^®^) and gefitinib (Iressa^®^, ZD1839), suppressed proliferation and induced apoptosis of the CD133^+^ fractions in three CSC lines established from human gliomas [74]. Therefore, despite specific mechanisms by which EGFR targeted therapies affect CSC being little understood, one may expect that the complete abrogation of EGF should affect the physiology of CSC.

Interestingly, the presence of growth factors has been linked to a decreased radiosensitivity in normal culture conditions of tumor cells [80,81]. In a study done by Wollman *et al.* on MCF-7 human breast cancer cells, under hormonal deprivation, more than 90% of the tumor cells were arrested in G_0_/G_1_ phase and underwent apoptosis after culture irradiation [80]. In contrast, the addition of EGF resulted in growth stimulation; increased percentage of cells in the S-phase of the cell cycle; increased radioresistance compared with controls; and increased cellular gluthatione (GSH) levels. Subsequently, the addition of a monoclonal antibody against EGFR (mAb-225) blocked the ability of EGF to enhance growth and mediate radioresistance [80]. Therefore, it is well expected that the castration of growth factors, such as EGF, might affect CSC growth not only directly by molecular targeted mechanisms, but also by abrogating the protective effect of such factors on radiation resistance.

### 4.2. Therapies Targeting CSC via Developmental Stem Cell Pathways

It is likely that an increased signaling through pathways that direct the differentiation of normal stem cells, such as the *Wnt*/*β*-catenin and Notch pathways, may contribute to radiation resistance, thus representing another attractive approach to target CSC [82]. In breast cancer cell lines for example, the overexpression of *β*-catenin in the stem-cell antigen-positive cells has shown to increase the ability of these cells to form mammospheres in culture, supporting a role in radiation resistance [83]. Moreover, the *Wnt*/*β*-catenin pathway may promote genomic instability after irradiation, thus allowing tumor cells to survive after irradiation. For that reason, *Wnt* inhibitors such as monoclonal antibodies against *Wnt*-1 and *Wnt*-2 have been designed for its evaluation in patients [84].

There is large evidence that the Notch pathway is dysregulated in a substantial amount of human cancers [85]. Notch signaling is activated in both breast CSC [86] and in endothelial cells [87] in response to radiation. Especially in breast cancer, a key role in the self-renewal of breast CSC has been ascribed to Notch signaling. For that reason, several approaches have been developed to inhibit Notch signaling. The most advanced clinically, is the development of γ secretase inhibitors. Activation of Notch signaling depends on the proteolytic activity of γ secretase, which cleaves Notch receptors releasing their intracellular domains. Inhibition of Notch signaling via γ-secretase inhibitors can potentially block CSC self-renewal and decrease medulloblastoma growth [88]. Currently available Notch signaling inhibitors include MK-0752, a γ-secretase inhibitor that is in clinical development for the treatment of advanced pancreatic cancer (ClinicalTrials.gov, number NCT01098344).

Another approach targeting developmental pathways that regulate the self-renewal of normal stem cells is based on the inhibition of the mammalian target of rapamycin (mTOR). The mTOR is considered a key signaling molecule that drives uncontrolled GBM tumor proliferation, and seems to be critical for breast CSC survival [89]. This serine-threonine kinase functions downstream from Akt in the phosphatidylinositol 3′-kinase/Akt/mTOR signaling pathway, regulating cellular growth and G_1_/S cell cycle progression [90]. Remarkably, the phosphatidylinositol 3′-kinase/Akt/mTOR signaling pathway is commonly activated in GBM through constitutive activation of upstream receptor tyrosine kinases, such as EGFR, and/or loss of PTEN tumor suppressor function [91,92]. Interestingly, a study published before the isolation of CSC from human GBM, demonstrated that rapamycin may enhance the efficacy of fractionated radiation of established U87 xenografts in nude mice, and such effect is closely associated to the inhibition of G_1_-specific cyclin-dependent kinase activity [93].

Interestingly, a small molecule specifically targeting the mTOR, has shown to eradicate leukemia-initiating cells and not normal stem or progenitor cells [94]. Furthermore, this drug was able to restore the normal hematopoietic stem cell function, which was impaired through disruption of PTEN molecule, which is localized upstream of mTOR [94]. In a similar study, the administration of rapamycin alone resulted in a significant decrease of CD133^+^ pancreatic CSC [95]. Immunohistochemical studies for detecting the expression of phospho-s6-ribosomal protein, which is located downstream of mTOR, confirmed that the pathway is only active in a small subset of cells, including the CSC population, and can be inhibited by rapamycin [95].

Another developmental pathway that regulates cancer stem cells is the Hedgehog pathway. This pathway may function directly in tumor cells as well as in the tumor stroma [96]. The activation of Hedgehog pathway has been reported to regulate cell proliferation through the activation of cyclins and cyclin-dependent kinases [97]. In addition, a complex role of the Hedgehog pathway has been suggested in the control of cell differentiation, and may involve the production of secreted proteins such as neurotrophic and angiogenic factors [98]. Small molecule inhibitors of the Hedgehog pathway are currently in early clinical studies being evaluated for safety and optimal dose/schedules, with proof of concept being explored [99,100]. In this regard, a phase I clinical trial conducted in patients with metastatic or locally advanced basal-cell carcinoma, has been recently published [99]. That study evaluated the safety and pharmacokinetics of GDC-0449, a small molecule inhibitor of smoothened homologue, and tumor responses in 33 patients. General conclusions of that study supported the use of GDC-0449 in locally advanced or metastatic basal-cell carcinoma patients [99]. Moreover, first phase II clinical trials utilizing Hedgehog inhibitor GDC-0449 alone (ClinicalTrials.gov, number NCT01201915) or in combination with cytotoxic agents and bevacizumab are ongoing (ClinicalTrials.gov, number NCT00636610).

Altogether, the above mentioned findings are encouraging and likely support the notion that it may become possible to solely target CSC, sparing normal stem cells, through the use of inhibitors that specifically target developmental pathways that regulate the self-renewal of normal stem cells. An update of clinical trials assessing these targets is illustrated in Table 1 [65].

### 4.3. Therapies Targeting CSC Microenvironment: The VEGF Molecule

There is also an increasing interest in the possibility of exploiting the putative CSC niche for drug targeting. This approach comes from the hypothesis that CSC may eventually dictate expansion of the normal niche, leading to an altered niche as the cells become independent of normal regulatory signals [56]. In line with this, it has been hypothesized that signals coming from an aberrant vascular niche mimicking the normal stem cells niche might be a way to sustain GBM CSC. Several experimental findings may support this hypothesis. Parallel findings by two separate groups have shown that freshly isolated CD133^+^ CSC-enriched cells, but not CD133^−^ glioblastoma cells, formed highly vascular tumors in the brains of immunocompromised mice [19,56]. Additionally, treatment of CD133^+^ cells with bevacizumab markedly inhibited their ability to initiate tumors *in vivo* and depleted both blood vessels and self-renewing CD133^+^ cells from tumor xenografts [56]. These studies confirmed earlier observations postulating that malignant gliomas are highly dependent on angiogenesis for its growth and maintenance and supported a key role for CSC in promoting tumor angiogenesis [101]. In the above mentioned study, the authors examined the potential of the so-called “stem cell-like glioma cells”, defined by the presence of the CD133 marker, to support tumor angiogenesis by xenografting human glioblastoma biopsy specimens into the brains of immunocompromised mice [19]. Interestingly, tumors from CD133^+^ cells were morphologically different from those formed by inoculation of CD133^−^ cells, due to widespread tumor angiogenesis, necrosis and hemorrhage. In addition, CD133^+^ cells secreted higher levels of VEGF leading to increase endothelial cell migration and tube formation *in vitro*, in comparison with matched-CD133^−^ cells. Finally, and in line with findings from Calabrese *et al.* [56], the VEGF-neutralizing antibody bevacizumab showed potent antiangiogenic activity *in vivo* and suppressed growth of xenografts derived from CD133^+^ cells, but limited efficacy in xenografts derived from matched CD133^−^ populations [19]. Altogether, these findings may support the use of antiangiogenic approaches as a valid strategy for targeting CSC populations in cancer treatment.

However, despite CSC and angiogenesis likely acting in parallel to promote tumor development and maintenance—which represents a promising approach for the design of new anticancer therapies— a deeper understanding of the relationship between CSC and vascular endothelium is critical. Remarkably, a recent paper has described the existence of a subpopulation of endothelial cells within GBM, harboring a CSC phenotype (CD133^+^/CD144^−^) and with the ability to differentiate into tumor and endothelial lineages, possibly via an intermediate CD133^+^/CD144^+^ progenitor cell [102]. Interestingly, the exposure of CD133^+^/CD144^−^ cells to bevacizumab had no impact on its ability to differentiate into endothelial progenitors, yet blocking further maturation from CD133^+^/CD144^−^ cells into CD105^+^ endothelial cells. This mechanism of action suggests that blocking VEGF inhibits the maturation of tumor endothelial progenitors, but does not abrogate the capacity of CD133^+^ CSC to differentiate into endothelial progenitors. Additionally, this novel finding highlights the capacity of the putative CSC to generate its own tumor vasculature providing a plausible explanation to the failure of antiangiogenic approaches currently in use in the clinic.

### 4.3. Combinatorial Approaches to Target CSC: Targeting the EGFR and Tumor Microenvironment

Molecular pathways involved in survival and replication of CSC are extremely complex, thus interfering with single steps in these pathways is expected to be an insufficient therapeutic approach. This complexity supports the need to interfere at different stages to avoid escape mechanisms of the tumor, and warrants a field for applying combinatorial studies. Considering this premise, and based on the identification of molecular mediators of therapeutic resistance in CSC, the first combinatorial clinical trials to evaluate potential synergistic benefits of adding CSC-targeted therapies to traditional anti-cancer regimens are being assessed.

One example is the γ secretase inhibitor RO4929097, which is undergoing clinical evaluation in different solid malignancies, such as advanced colorectal cancer and breast cancer. A first phase II study with RO4929097, as neoadjuvant therapy, in combination with the anti-EGFR monoclonal antibody cetuximab, has started with the goal of evaluating the ability of RO4929097 to abrogate primary cetuximab resistance in patients with advanced colorectal cancer bearing a k-*ras* mutation (ClinicalTrials.gov, number NCT01198535). A second phase II trial aiming at evaluating RO4929097 as neoadjuvant therapy in combined chemotherapy and bevacizumab, has started for treating patients with advanced colorectal cancer (ClinicalTrials.gov, number NCT01270438). Moreover, a phase I clinical trial is ongoing to evaluate the side effects and best dose of giving the Hedgehog inhibitor GDC-0449 together with RO4929097 in women with advanced breast cancer (ClinicalTrials.gov, number NCT01071564).

Although the mechanisms underlying chemo- and radioresistance of CSC are not fully understood, it has been well demonstrated that this resistance is responsible, at least in part, to the low efficacy of traditional anti-cancer therapies [103]. Therefore, the combination of agents targeting CSC-pathways with cytotoxic chemotherapy and/or radiation therapy may provide the means for the eradication of both CSC and bulk tumor cell populations, warranting its evaluation in clinical studies. A phase II trial aimed at evaluating the γ secretase inhibitor MK-0752 as neoadjuvant therapy in combination with tamoxifen or the aromatase inhibitor letrozole has started for treating patients with early, estrogen receptor-positive breast cancer. Other examples of such combination studies have been mentioned previously.

Despite not having reached a similar development stage to the aforementioned therapies against stem cells-specific pathways, other approaches have entered early clinical evaluation, such as direct ablation by targeting key molecules (e.g., EGFR) and indirect strategies (e.g., antiangiogenic therapy).

The relationship between EGFR signaling and VEGF is a representative example of the cross-stimulation among different molecular pathways [104-108]. EGFR and VEGF share common downstream signaling pathways, and combining drugs that target these molecules may confer additional clinical benefits [109]. In fact, several drugs that were originally developed as EGFR blocking agents (e.g., erlotinib, cetuximab and nimotuzumab) have shown an inhibitory effect on angiogenesis by blocking the VEGFR or by inhibiting the secretion of angiogenic growth factors [75].

In addition, there is an increasing body of evidences associating the role of CSC in the stimulation of angiogenesis, which in turn may promote tumor growth, increase tumor aggressiveness and mediate resistance to conventional therapies. In fact; recent reports have illustrated how a close interplay between CSC, angiogenesis, and the tumor vasculature may impair the efficacy of radiotherapy [57]. In glioma, for example, irradiated CSC-derived tumors are particularly vascularized and hemorrhagic [19], suggesting that endothelial cell survival after irradiation may contribute to angiogenesis and tumor growth [57]. Moreover, *in vivo* administration of nimotuzumab significantly reduced the growth and vascularity of brain tumor xenografts, while inhibiting the tumor invasiveness promoted by irradiation, acting as a radiosensitizer [73]. Remarkably, whereas earlier studies indicated that VEGF is induced in tumors after irradiation [110], nimotuzumab downregulated VEGF expression in tumor xenografts [75]. Therefore, it is well expected that mutual blockade through therapies directed against both endothelial cells and tumor cells may affect CSC-mediated tumor growth by disrupting the vascular endothelial microenvironment, in addition to abrogate the EGFR-mediated activation on these cells.

## 5. Conclusions

In summary, preliminary evidence of novel therapies against CSC are encouraging, and suggest that specific therapies against CSC are plausible, opening a new era in cancer research and treatment. Nevertheless, although therapies against CSC are likely to offer an exciting promise of treatment for cancer therapy, we must be cautious with their application, due to their potential side effects. New CSC-oriented therapies should be able to kill CSC, avoiding or minimizing the normal tissue stem cell toxicity. For that reason, the appropriate identification of CSC-specific targets that are not vital to normal tissue stem cells is mandatory. New studies should compare CSC gene expression profiles and functional properties with those of relevant normal stem cells, an issue that might lead us to a more refined identification of CSC populations that predict resistance to conventional therapies and truly correlate the presence of CSC with clinical outcome of patients. Validating the presence of well documented targets in cancer treatment, such as the EGFR, in the CSC population should be further explored in the development of the new anti-CSC therapies, taking into consideration the direct correlation between the levels of expression of EGFR and the biologic activity of some EGFR antagonists. Additionally, given the accumulated evidence for CSC dependence of tumor vasculature, combining cytotoxic therapies with antiangiogenic approaches may mediate the targeted anti-CSC effects in order to provide long-term disease-free survival in patients.

## Figures and Tables

**Table 1.** Update on clinical trials for CSC molecular targets.

**Target**	**Drug**	**Type of cancer**	**Phase**	http://clinicaltrials.gov/ [65] **Identifier**	**Other agents**	**Estimated Enrollment**	**Estimated study completion date**
**Notch**	MK0752	Pancreatic	I, II	NCT01098344	Gemcitabine/Radiation	60	December, 2014
		Breast	I	NCT00106145		50	May, 2011
	RO4929097	Renal cell	II	NCT01141569		39	June, 2011
		Colorectal	II	NCT01198535	Cetuximab	132	October, 2013
		Colorectal	II	NCT01270438	Bevacizumab/FOLFOX	98	April, 2011
**Hedgehog**	IPI-926	HN	I	NCT01255800	Cetuximab	24	March, 2013
	GDC-0449	Colorectal	II	NCT00636610	Bevacizumab/QT	198	March,2011
		BCC	II	NCT01201915		49	November, 2012
**Hedgehog/ Notch**		Breast	I	NCT01071564	RO4929097	36	September, 2010
	BMS-833923	BCC	I	NCT00670189		70	May, 2012
	PF-04449913	Hematologic	I	NCT00953758	Dasatinib or Bosutinib	94	June, 2013
**Wnt**	Resveratrol	Colon	I, II	NCT00256334		12	December, 2009
	PRI-724	Solid tumors	I	NCT01302405		64	March, 2013

*NOTE*: As listed in clinicaltrials.gov on May 12, 2011; Abbreviations: BCC: basal cell carcinoma; HN: head and neck; QT: chemotherapy.
